# Fauna of some families of Coleoptera (Insecta) in the Republic of Mordovia (Russia)

**DOI:** 10.3897/BDJ.12.e117041

**Published:** 2024-02-06

**Authors:** L.V. Egorov, Alexander Ruchin, Sergei K. Alekseev, Oleg Artaev, Evgeniy A. Lobachev, Sergei V. Lukiyanov, Gennadiy B. Semishin

**Affiliations:** 1 The State Nature Reserve «Prisursky», Cheboksary, Russia The State Nature Reserve «Prisursky» Cheboksary Russia; 2 Joint Directorate of the Mordovia State Nature Reserve and National Park «Smolny», Saransk, Russia Joint Directorate of the Mordovia State Nature Reserve and National Park «Smolny» Saransk Russia; 3 Ecological club "Stenus", Kaluga, Russia Ecological club "Stenus" Kaluga Russia; 4 Papanin Institute for Biology of Inland Waters Russian Academy of Sciences, Borok, Russia Papanin Institute for Biology of Inland Waters Russian Academy of Sciences Borok Russia; 5 National Research Mordovia State University, Saransk, Russia National Research Mordovia State University Saransk Russia

**Keywords:** dataset, faunistic records, data paper, Elateridae, Cantharoidea, Cerambycidae, Coccinellidae, Drilidae, Lycidae, Lampyridae

## Abstract

**Background:**

Biodiversity conservation is an important goal of most ecosystem management efforts. Therefore, proper monitoring of biodiversity requires constant attention. Coleoptera should be monitored as an essential part of the overall biodiversity. Special monitoring is needed for families that are active as predators (e.g. Coccinellidae) or are saproxylic species (e.g. Elateridae and Cerambycidae). The aim of the research is to describe the fauna of seven families of Coleoptera (Elateridae, Drilidae, Lycidae, Lampyridae, Cantharidae, Coccinellidae and Cerambycidae) of the Republic of Mordovia (the centre of the European part of Russia). The results are based on faunistic research, the main part of which was carried out in April-October 2007-2023 and on material from museum collections. The collecting was made using several different methods (by hand, light trapping, on different lures, into pitfall traps etc.). GPS coordinates are given for each faunistic record.

**New information:**

The dataset contains information on seven species new to the region: *Malthodesflavoguttatus* Kiesenwetter, 1852, *Malthodesminimus* (Linnaeus, 1758) (Cantharidae); *Scymnusrubromaculatus* (Goeze, 1777) (Coccinellidae); *Anoploderarufipesventralis* Heyden, 1886, *Tragosomadepsarium* (Linnaeus, 1767), *Xylotrechusarvicola* (Olivier, 1795) and *Xylotrechusibex* (Gebler, 1825) (Cerambycidae).

## Introduction

Current knowledge of global biodiversity is based on the extrapolation of small samples to total species richness (Sánchez-Bayo and Wyckhuys 2019, Ronquist et al. 2020, Fathima et al. 2021, Caminha-Paiva et al. 2022). However, tropical regions and biodiversity hotspots are popular inventory targets due to their impressive species richness (Lister and Garcia 2018). Studies of local insect faunas of the temperate zone are also available (e.g. Babytskiy et al. (2019), Kwon et al. (2020), Nunes et al. (2020), Barkalov and Khruleva (2021), Polevoi (2021)). In recent years, there has been a need to document and understand nature as fast as possible to provide us with an informed systemic response to the accelerating impact that humanity is having on ecosystems (Essl et al. 2015). The volume of biodiversity data is growing rapidly and records in various biodiversity databases are constantly being updated (La Salle et al. 2016). The purpose of any faunal research is to register species in a certain locality. Additionally, such intensive faunal studies are necessary if we want to determine species richness on a local scale and monitor long-term changes in species diversity (Ejsmont‐Karabin 2019).

Coleoptera are considered to be the most taxonomically diverse group of insects, which includes the main components of ecosystems in terms of biomass, species richness and ecological role (Stack 2015). About 360,000 species have been described (Bouchard et al. 2009), which, according to some estimates, is about 25% of the total diversity of animals on Earth (Hunt et al. 2007). Beetles play important roles in pollination, utilisation of organic matter, predator-prey interactions and soil disturbance. Some beetle families are used as indicators of the state of ecosystems or to determine the species richness of different regions (Huffaker and Gutierrez 1999, Ruchin et al. 2019a, Avtaeva et al. 2021, Sundukov and Makarov 2021). For example, Cerambycidae, Curculionidae and Elateridae have been identified as "useful surrogates" for Coleoptera diversity in forest ecosystems (Ohsawa 2010, Zamoroka 2022).

The intensification of research on the Coleoptera fauna in the Republic of Mordovia has made it possible to publish lists of species from various families. Thus, information was given on the species diversity of Coccinellidae (Ruchin et al. 2019b, Ruchin et al. 2020b), Cerambycidae (Ruchin and Egorov 2018a, Ruchin and Egorov 2018b), Elateridae (Ruchin et al. 2018) and Cantharoidea (Kazantsev et al. 2019, Ruchin and Egorov 2019). Previously, information about the Coleoptero fauna of the Mordovia State Nature Reserve was summarised (Egorov et al. 2020). However, these lists did not specify clear coordinates of the places of findings that would allow us to accurately describe the distribution of the species. In this regard, we have created a dataset that includes descriptions of findings of species from these groups of Coleoptera (Egorov et al. 2023).

## General description

### Additional information

Each observation includes basic information, such as location (latitude/longitude), date of observation, observer name and identifier name. Coordinates were determined in the field using a GPS device or, after surveys, using Google Maps. A total of 14,712 specimens were studied.

The dataset contains data on 256 species of Coleoptera from seven families found in the territory of the Republic of Mordovia: Elateridae (62 species), Drilidae (1 species), Lycidae (8 species), Lampyridae (1 species), Cantharidae (30 species), Coccinellidae (48 species) and Cerambycidae (106 species) (Table 2). In addition, seven more species are known from other publications, which, however, we did not encounter during our research: these are Elateridae (2 species), Cantharidae (1 species), Coccinellidae (2 species) and Cerambycidae (2 species) (Table 1). Therefore, the total list of the fauna of these families includes 263 species.

The dataset also contains information on seven species new to the region: *Malthodesflavoguttatus* Kiesenwetter, 1852, *Malthodesminimus* (Linnaeus, 1758) (Cantharidae); *Scymnusrubromaculatus* (Goeze, 1778) (Coccinellidae); *Anoploderarufipesventralis* Heyden, 1886, *Tragosomadepsarium* (Linnaeus, 1767), *Xylotrechusarvicola* (G.-A. Olivier, 1800) and *Xylotrechusibex* (Gebler, 1825) (Cerambycidae). The occurrence of most of these taxa in Mordovia was to be expected. The most interesting is the finding of two species of Cerambycidae. The find of *Tragosomadepsarium* is one of the southernmost findings of the species in the European part of Russia (Danilevsky 2020, Anisimov and Bezborodov 2021). Mordovia is probably part of the southern limit of the species' range in Russia. This species is subject to protection in many European countries. It is found in forests with dead wood, where slight temperature fluctuations occur. In contrast, our finding or sighting of *Anoploderarufipesventralis* is one of the northernmost records for the species within the European part of Russia (in the western part, the species is known northwards to the Tula and Kaluga oblasts and in the eastern part, to the Samara and Ulyanovsk oblasts) (Danilevsky 2014).

According to the number of individuals in the dataset, the families Cerambycidae and Elateridae are the richest in species diversity. Species from the families Drilidae and Lycidae were found only once. Most species of these families are secretive and difficult to find in natural habitats. The only abundant representative of Lycidae was *Lygistopterussanguineus* (Linnaeus, 1758), which is an anthophilous species and very common in various ecosystems of the region.

To compare biodiversity, Table 2 was compiled. The number of species of the seven families differs by regions of European Russia, which is due to the different degree of study of the Coleoptera fauna of a particular region. The fauna of the Moscow oblast is the most studied (Nikitsky 2019).

## Sampling methods

### Sampling description

We used traditional methods of collecting ground beetles, including manual collection, pitfall traps, Malaise traps, window traps, pan traps, light traps and trapping with various baits in 2002–2023 (Golub et al. 2012, Ruchin et al. 2020). Pitfall traps were installed during April–October 2007–2009 and 2012–2022. The traps were 0.5-litre plastic cups containing 200 ml of a 4% formalin solution. Malaise traps were installed one trap at a time in different biotopes in 2021–2022. Pan traps were used more often in open biotopes (on the forest edges, clearings and meadows). At the same time, from 5 to 10 pan traps were installed in one row in 2020–2023. Window traps were used to survey areas of cluttered and old-growth forests in 2014–2022. The bait trapping used fermented beer with sugar, vinegar and rotten meat. The dataset also includes information from the collections of the Mordovia State Nature Reserve from the years 1971–1975, 1984 and 1988.

### Quality control

The classification of taxa into families is based on the modern works of McKenna et al. (2019) and Cai et al. (2022). The species lists have been checked according to the Catalogue of Palaearctic Coleoptera (Löbl and Smetana 2007, Danilevsky 2020), as well as Robertson et al. (2015). The GPS coordinates of the records before 2007 were obtained using Google Maps web application and after 2007, using geopositioning devices.

## Geographic coverage

### Description

**Figure 1.**
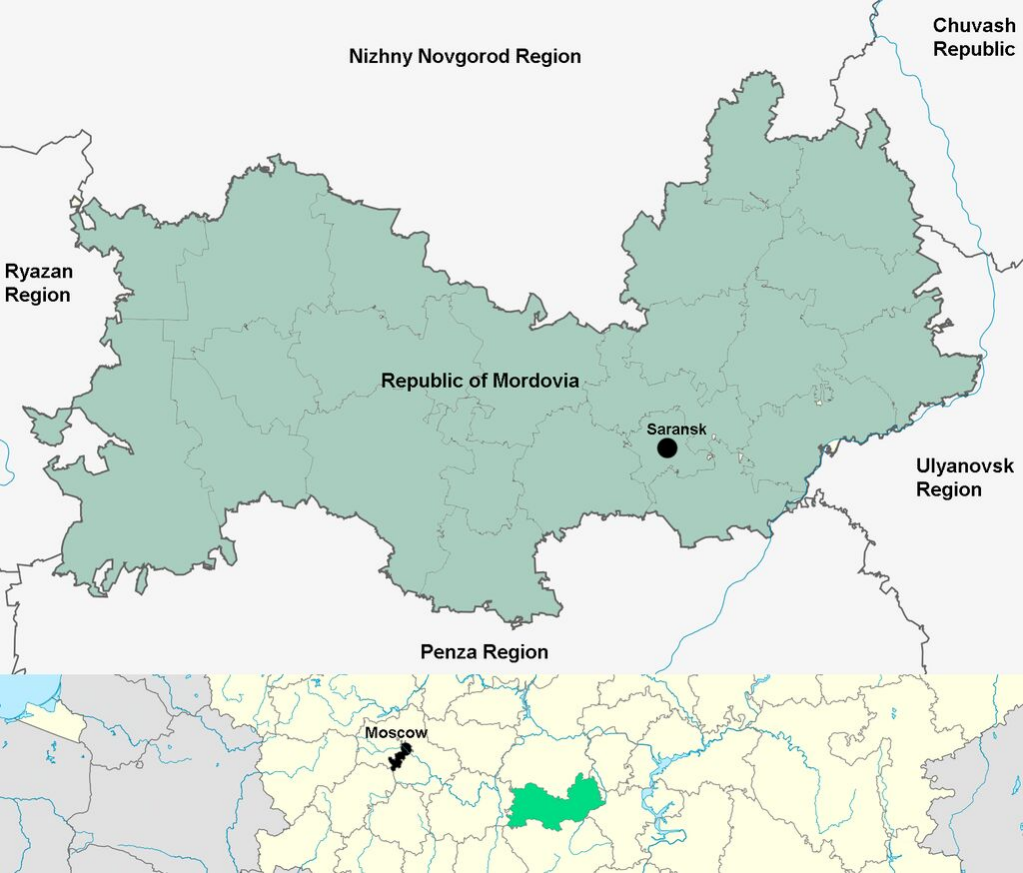
Study area.

The Republic of Mordovia is located in the centre of the Russian Plain between 42°11'E and 46°45'E longitude and 53°38'N and 55°11'N latitude in the interfluve of the Moksha and Sura Rivers (the Volga River Basin). The region lies within the Volga Upland (eastern part) and the Oka-Don Lowland (western part). The Sura River (right tributary of the Volga) flows along the south-eastern border, its main tributaries being the Alatyr, Cheberchinka, Shtyrma and Mena rivers. The Moksha River (a right tributary of the Oka River) flows along the western part of the region; its basins include the Rivers Vad, Sivin, Issa, Satis, Urey and Urkat. The area of the Republic of Mordovia is 26,121 km^2^. The territory is part of the temperate climate zone with a well-defined change of seasons. The maximum length from west to east is 298 kilometres and from north to south, up to 140 kilometres (Fig. 1). The position of the Republic in the sector of moderate continental climate causes instability of humidification: wet years alternate with dry years (Yamashkin et al. 2004). The nature of the Republic is characterised by high landscape diversity (Chursina and Ruchin 2018). In the structure of soil cover, there is a combination of sod-podzolic, grey forest soils and chernozems. The natural vegetation is dominated by pine forests with an admixture of spruce, oak forests and meadow steppes. The dominance of forest-steppe landscapes favours the development of agriculture (Yamashkin 1998).

### Coordinates

53.64 and 55.19 Latitude; 42.16 and 46.73 Longitude.

## Usage licence

### Usage licence

Creative Commons Public Domain Waiver (CC-Zero)

### IP rights notes

CC BY 4.0

## Data resources

### Data package title

Biodiversity of the families Elateridae, Drilidae, Lycidae, Lampyridae, Cantharidae, Cerambycidae and Coccinellidae (Coleoptera, Insecta) in Republic of Mordovia (Russia).

### Resource link

https://doi.org/10.15468/gj38hk

### Alternative identifiers

https://www.gbif.org/dataset/905111e8-a738-48e8-9a90-09431e4b6a2c

### Number of data sets

1

### Data set 1.

#### Data set name

Biodiversity of the families Elateridae, Drilidae, Lycidae, Lampyridae, Cantharidae, Cerambycidae and Coccinellidae (Coleoptera, Insecta) in Republic of Mordovia (Russia).

#### Data format

Darwin Core

#### Character set

UTF-8

#### Download URL

http://gbif.ru:8080/ipt/archive.do?r=2023_mgpz_coleopt

#### Description

The dataset contains data on 7,689 occurrences of 256 species of seven families of the order Coleoptera in the territory of Mordovia (Russia) from 1972–2023.

**Data set 1.** 

Column label	Column description
occurrenceID	An identifier for the Occurrence (as opposed to a particular digital record of the occurrence).
basisOfRecord	The specific nature of the data record: HumanObservation.
scientificName	The full scientific name including the genus name and the lowest level of taxonomic rank with the authority.
kingdom	The full scientific name of the kingdom in which the taxon is classified.
phylum	The full scientific name of the phylum or division in which the taxon is classified.
class	The full scientific name of the class in which the taxon is classified.
order	The full scientific name of the order in which the taxon is classified.
taxonRank	The taxonomic rank of the most specific name in the scientificName.
decimalLatitude	The geographic latitude of location in decimal degree
decimalLongitude	The geographic longitude of location in decimal degrees.
coordinateUncertaintyInMeters	The horizontal distance (in metres) from the given decimalLatitude and decimal-Longitude describing the smallest circle containing the whole of the Location.
geodeticDatum	The ellipsoid, geodetic datum or spatial reference system (SRS), upon which the geographic coordinates given in decimalLatitude and decimalLongitude are based.
country	The name of the country in which the Location occurs. Here - Russia.
countryCode	The standard code for the country in which the Location occurs. Here - RU.
individualCount	The number of individuals represented present at the time of the Occurrence.
eventDate	The date when material from the trap was collected or the range of dates during which the trap collected material.
year	The integer day of the month on which the Event occurred.
month	The ordinal month in which the Event occurred.
day	The integer day of the month on which the Event occurred.
recordedBy	A person, group or organisation responsible for recording the original Occurrence.
identifiedBy	A list of names of people, who assigned the Taxon to the subject.
locality	The original textual description of the place.
georeferenceSources	A maps service used to georeference the location.

## Figures and Tables

**Table 1.** Number of Coleoptera species from eight families found in the territory of the Republic of Mordovia. * – data from publications were used (Ruchin and Egorov 2018a, Ruchin and Egorov 2018b, Ruchin and Egorov 2019). ** - not found in our studies, but these species are found in the nearest regions and may inhabit the Republic of Mordovia. *** - the species is absent from our studies and studies in neighbouring regions, occurrences from publications is not confirmed by collection materials.

**Family**	**Number of species in the dataset**	Number of species in the region*	Species not included in the dataset**	Species that are excluded from the fauna lists***
Elateridae	62	64	*Melanotusbrunnipes* (Germar, 1823), *Orithaleserraticornis* (Paykull, 1800)	*Agriotespilosellus* (Schönherr, 1817), *Melanotuscrassicollis* (Erichson, 1841), *Melanotusfusciceps* (Gyllenhal, 1817), *Liotrichusaffinis* (Paykull, 1800), *Stenagostusrufus* (De Geer, 1774), *Pseudanostirusglobicollis* (Germar, 1843)
Drilidae	1	1		
Lycidae	8	8		
Lampyridae	1	1		
Cantharidae	30	31	*Cantharisnigra* (De Geer, 1774)	*Rhagonychafemoralis* (Brulle, 1832)
Coccinellidae	48	50	*Hippodamiaseptemmaculata* (De Geer, 1775), *Coccinellaundecimpunctata* Linnaeus, 1758	*Coccinulasinuatomarginata* (Faldermann, 1837), *Parexochomusnigromaculatus* (Goeze, 1777)
Cerambycidae	106	108	*Anaesthetistestacea* (Fabricius, 1781), *Monochamussaltuariusoccidentalis* Sláma, 2017	*Anastrangaliadubia* (Scopoli, 1763), *Aromiamoschataambrosiaca* (Steven, 1809), *Brachytavariabilis* (Gebler, 1817), *Ergatesfaber* (Linnaeus, 1761), *Rhagiumbifasciatum* Fabricius, 1775, *Stenurellajaegeri* (Hummel, 1825), *Stictolepturafulva* (De Geer, 1775)
**Total**	256	263	7	16

**Table 2.** Comparison of biodiversity of several regions of Russia with well-studied fauna within the families under study (comparative data).

**Family**	**Republic of Mordovia**	**Mos cow oblast**	**Chuvash Republic**	**Samara oblast**	**Udmurt Republic**	**Lipetsk oblast**	**Voronezh oblast**
Elateridae	64	69	67	50	63	52	63
Drilidae	1	7	1	1	0	1	1
Lycidae	8	7	6	3	6	4	3
Lampyridae	1	2	1	1	1	1	1
Cantharidae	31	48	30	21	27	32	23
Coccinellidae	50	63	52	53	47	52	53
Cerambycidae	108	129	113	157	110	92	110
**Total**	**263**	**320**	**2 70**	**286**	**2 54**	**234**	**254**
Data	Our data	(Nikitsky 2019)	Data of the first author	(Rosenberg 2007); data of A.S. Tilly and D.V. Magdeev	(Dedyukhin et al. 2005); data of S.V. Dedyukhin	(Mazurov et al. 2022)	(Negrobov 2005); data of A.A. Prokin and A.S. Sazhnev
